# Octameric structure of *Staphylococcus aureus* enolase in complex with phosphoenolpyruvate

**DOI:** 10.1107/S1399004715018830

**Published:** 2015-11-26

**Authors:** Yunfei Wu, Chengliang Wang, Shenglong Lin, Minhao Wu, Lu Han, Changlin Tian, Xuan Zhang, Jianye Zang

**Affiliations:** a Hefei National Laboratory for Physical Sciences at Microscale and School of Life Sciences, Collaborative Innovation Center of Chemistry for Life Science, University of Science and Technology of China, 96 Jinzhai Road, Hefei, Anhui 230026, People’s Republic of China; b Key Laboratory of Structural Biology, Chinese Academy of Sciences, Hefei, Anhui 230027, People’s Republic of China; c High Magnetic Field Laboratory, Hefei Institutes of Physical Science, Chinese Academy of Sciences, Hefei, Anhui 230031, People’s Republic of China; d National Synchrotron Radiation Laboratory, University of Science and Technology of China, Hefei, Anhui 230027, People’s Republic of China

**Keywords:** enolase, *Staphylococcus aureus*, complex structure, octamerization

## Abstract

*Staphylococcus aureus* is a Gram-positive bacterium with strong pathogenicity that causes a wide range of infections and diseases. Enolase is an evolutionarily conserved enzyme that plays a key role in energy production through glycolysis. Additionally, enolase is located on the surface of *S. aureus* and is involved in processes leading to infection. Here, crystal structures of *Sa_*enolase with and without bound phosphoenolpyruvate (PEP) are presented at 1.6 and 2.45 Å resolution, respectively. The structure reveals an octameric arrangement; however, both dimeric and octameric conformations were observed in solution. Furthermore, enzyme-activity assays show that only the octameric variant is catalytically active. Biochemical and structural studies indicate that the octameric form of *Sa*_enolase is enzymatically active *in vitro* and likely also *in vivo*, while the dimeric form is catalytically inactive and may be involved in other biological processes.

## Abstract 2

Non-ligand-bound and PEP-bound structures of *S. aureus* enolase (*Sa*_enolase) were solved, and catalytic loop 1 in the PEP-bound structure was found to show both ‘open’ and ‘closed’ conformations. Structural and biochemical results indicate that octamerization is required for substrate binding and catalysis by *Sa*_enolase.

## 1. Introduction   

*Staphylococcus aureus* is a human pathogen that causes a wide range of infections. Over the past century, *S. aureus* has been implicated in infections occurring both inside and outside hospitals and continues to be a cause of concern (Otto, 2012 ▸). A variety of clinical diseases are caused by *S. aureus* infection, including fasciitis, pneumonia, endocarditis, septicaemia, osteomyelitis and toxic shock syndrome, with morbidity and mortality rates dependent upon individual cases (Ansari *et al.*, 2014 ▸; Shopsin & Kreiswirth, 2001 ▸).

The pathogenic mechanism and host immunity response associated with *S. aureus* infections are ambiguous (Proctor, 2012 ▸) and there is little prospect for a universal vaccine, given the extensive genetic and antigenic variability (Pier, 2013 ▸). A number of *S. aureus* virulence factors comprising exotoxins, extracellular enzymes and cell-surface proteins have been demonstrated to play key roles in host-cell adherence and invasion (Lina *et al.*, 1999 ▸; Liu *et al.*, 2005 ▸; Santala *et al.*, 1999 ▸; Tong *et al.*, 2012 ▸). Interestingly, enolase has been identified on the cell surface of *S. aureus*, enhancing the staphylokinase activation of plasminogen (Mölkänen *et al.*, 2002 ▸). Enolase is also capable of binding laminin, which is abundant in the extracellular matrix and is involved in pathogen invasion (Carneiro *et al.*, 2004 ▸).

Enolase is a ubiquitous enzyme that is found in all living organisms and is a member of the enolase superfamily, which includes muconate-lactonizing enzyme and mandelate racemase (Gerlt *et al.*, 2005 ▸). Enolase is a glycolytic enzyme that requires a divalent metal ion (optimally Mg^2+^) bound in the active site (Gerlt *et al.*, 2011 ▸) to catalyze the dehydration of 2-phosphoglycerate (2-PG) to phosphoenolpyruvate (PEP), as well as the reverse reaction in the gluconeogenesis pathway (Babbitt *et al.*, 1996 ▸). Despite the absence of a signalling sequence, localization of enolase to the surface of prokaryotic and eukaryotic cells has been reported; however, the transport mechanism remains unknown (Seweryn *et al.*, 2007 ▸). Surface enolase has been confirmed as a plasminogen receptor in pathogenic streptococci (Feng *et al.*, 2009 ▸), haematopoietic cells, neuronal cells and endothelial cells (Fukano & Kimura, 2014 ▸), and is involved in regulating pericellular fibrinolytic activity and extracellular matrix degradation, enhancing monocyte migration and promoting cell migration in cancer metastasis (Capello *et al.*, 2011 ▸; Wygrecka *et al.*, 2009 ▸; Godier & Hunt, 2013 ▸). Additionally, eukaryotic enolase exhibits various subcellular localizations and functions related to pathophysiologies including cancer, apoptosis, arthritis and Alzheimer’s disease (Díaz-Ramos *et al.*, 2012 ▸). Furthermore, enolase is a member of the RNA degradosome, interacting with RNase Y in *Bacillus subtilis* (Newman *et al.*, 2012 ▸) and RNase E in *Escherichia coli* (Nurmohamed *et al.*, 2010 ▸).

The multiple functions of *Sa*_enolase encouraged further exploration, leading to determination of the crystal structure of *Sa*_enolase at 2.45 Å resolution. Moreover, the structure of *Sa*_enolase in complex with PEP was also determined at 1.6 Å resolution, enabling the elucidation of the interaction network between the enzyme and PEP. The activity assays reveal that the enzymatic activity of the dimeric and octameric variants fluctuates significantly and that the octameric form is a functional unit for catalytic activity *in vitro* and likely *in vivo*. Isothermal titration calorimetry (ITC) data show that the *Sa*_enolase dimer is unable to bind the substrate 2-PG. Results from continuous-wave electron paramagnetic resonance (CW-EPR) spectroscopy further indicate that the dynamic motion of the catalytic loop 1 (L1), the loop which is involved in substrate binding, is faster in the dimer than in the octamer. The addition of the substrate 2-PG is able to stabilize the L1 loop in the octamer while making no change in the dimer, correlating the quaternary structure with enzyme function.

## 2. Methods   

### 2.1. Cloning, expression and purification   

*Sa*_enolase (GenBank CEH25490.1) was amplified from the *S. aureus* genome and the amplified product was purified and excised *via* restriction-enzyme digestion using BamHI and XhoI and then inserted into a similarly digested pET-24a vector. The *Sa*_enolase plasmid was transformed into *E. coli* BL21(DE3) (Novagen) cells and grown overnight at 37°C in a 20 ml starter culture of Luria–Bertani (LB) medium containing 20 µg ml^−1^ kanamycin. The overnight starter culture was transferred into 1 l LB medium and incubated at 37°C to an OD_600_ of ∼0.6–0.8. The culture was induced with 0.2 m*M* isopropyl β-d-1-thiogalactopyranoside and incubated for ∼20 h at 16°C. The cells were harvested by centrifugation and lysed by sonication in a lysis buffer consisting of 50 m*M* Tris–HCl, 500 m*M* NaCl, 5 m*M* imidazole (IMD) pH 8.0. The C-terminally hexahistidine-tagged protein was first purified by nickel–nitriloacetic acid (Ni–NTA) affinity chromatography. The lysate underwent centrifugation at 23 800*g* for 30 min at 4°C and the resulting supernatant was subjected to a column containing 2 ml Ni–NTA resin (Qiagen) equilibrated with lysis buffer. The column was washed with 25 column volumes of buffer consisting of 50 m*M* Tris–HCl, 500 m*M* NaCl, 50 m*M* IMD pH 8.0 and the bound protein was recovered with 50 m*M* Tris–HCl, 500 m*M* NaCl, 250 m*M* IMD pH 8.0. The eluted samples were further purified using a HiLoad Superdex 200 16/60 (GE Healthcare) column with 20 m*M* HEPES, 150 m*M* NaCl pH 7.5, and the two peaks corresponding to octameric and dimeric forms of *Sa*_enolase were separated, collected and assessed by SDS–PAGE. The purified protein was concentrated to 10 mg ml^−1^ using 10 kDa molecular-weight cutoff filters (Millipore) in preparation for crystallization. Site-directed mutagenesis was performed using primers containing the desired mutation on plasmids containing the wild-type gene as the template and the amplified products were cloned into pET-22b vector. All plasmids were verified by sequence analysis. The mutant proteins were expressed and purified as described above.

### 2.2. Crystallization   

Crystals were grown at 16°C using the sitting-drop vapour-diffusion method with drops consisting of 1 µl protein solution (10 mg ml^−1^) mixed with an equal volume of reservoir solution. The initial crystallization conditions for non-ligand-bound *Sa*_enolase were determined using the Crystal Screen kit (Hampton Research). The optimized condition consisted of 0.01 *M* cobalt(II) chloride, 0.1 *M* MES pH 6.5, 1.8 *M* ammonium sulfate. Crystals of PEP-bound *Sa*_enolase were obtained by co-crystallization of *Sa*_enolase with 2-PG substrate at a protein:ligand molar ratio of 1:10. The initial crystallization conditions for PEP-bound *Sa*_enolase were determined using the Index kit (Hampton Research). The optimized condition consisted of 0.2 *M* ammonium acetate, 0.1 *M* Tris–HCl pH 8.5, 25%(*w*/*v*) polyethylene glycol 3350.

### 2.3. X-ray data collection and processing   

All crystals were soaked in cryoprotectant buffer containing 20%(*v*/*v*) glycerol and were then flash-cooled in liquid nitrogen. X-ray diffraction data were collected on the BL17U1 synchrotron-radiation beamline at Shanghai Synchrotron Radiation Facility (SSRF) using an ADSC Quantum 315r CCD detector with crystal-to-detector distances of 300 mm for non-ligand-bound *Sa*_enolase and 250 mm for PEP-bound *Sa*_enolase. Individual frames were collected at 100 K using 1 s for each 1.0° oscillation over a range of 200° for both data sets. Diffraction data were indexed, integrated, scaled and merged using *HKL*-2000 (Otwinowski & Minor, 1997 ▸).

### 2.4. Structure determination and refinement   

The *Sa*_enolase structure was determined by molecular replacement using *Phaser* (McCoy *et al.*, 2007 ▸) as implemented in the *CCP*4 package (Winn *et al.*, 2011 ▸). A monomer structure of *E. coli* enolase (PDB entry 1e9i; Kuhnel & Luisi, 2001 ▸) was used as the search model. After several rounds of refinement using *REFMAC*5 (Murshudov *et al.*, 2011 ▸) and *Coot* (Emsley & Cowtan, 2004 ▸), the structure of non-ligand-bound *Sa*_enolase was refined to 2.45 Å resolution with a final *R*_work_ of 18.55% (*R*_free_ = 22.86%). The structure of *Sa*_enolase in complex with PEP was determined by molecular replacement using the non-ligand-bound form of *Sa*_enolase as the model. The PEP-bound *Sa*_enolase structure was refined to 1.6 Å resolution with a final *R*_work_ of 14.84% (*R*_free_ = 16.35%). The final model quality was analyzed by *PROCHECK* (Laskowski *et al.*, 1993 ▸). The data-collection and structure-determination statistics are listed in Table 1 ▸. All structural figures were prepared using *PyMOL* (DeLano, 2002 ▸).

### 2.5. Enzyme-activity measurement assay   

Kinetic studies were performed at room temperature in 20 m*M* IMD–HCl, 400 m*M* KCl, 1 m*M* magnesium acetate pH 7.0 buffer with the addition of 15 n*M* *Sa*_enolase and varying concentrations of 2-PG (0.1–1 m*M*) in a final volume of 100 µl. The increase in the absorption peak at 240 nm, corresponding to the product PEP, was recorded at 50 s intervals for 5 min following the addition of 2-PG. Michaelis–Menten and Eadie–Hofstee plots were used to derive the kinetic parameters. Activity assays for *Sa*_enolase mutants were performed in the same buffer using 30 n*M* enzyme and 1 m*M* 2-PG in a final volume of 100 µl. The increase in product was recorded at 20 s intervals for 100 s following the addition of 2-PG. Linear fitting images were created using *Origin* 8 (MicroCal Inc.).

### 2.6. Isothermal titration calorimetry (ITC)   

Isothermal titration calorimetry (ITC) assays were performed at 20°C. Each measurement was carried out by injecting 40 µl 2-PG (1 m*M*) into a cell containing 275 µl (50 µ*M*) *Sa*_enolase protein sample (wild-type octamer, wild-type dimer, F139A mutant or D355A mutant) in 20 m*M* HEPES, 150 m*M* NaCl pH 7.5. All ITC results were fitted to a one-site binding model using *Origin* 8 (MicroCal Inc.).

### 2.7. Continuous-wave electron paramagnetic resonance (CW-EPR)   

T43C/C243S mutant *Sa*_enolase protein was constructed by site-directed mutagenesis using the wild-type plasmid as the template. The protein was expressed and purified in the same way as the wild type. The dimeric and octameric forms were separately collected and concentrated to 10 mg ml^−1^. Samples were immediately reacted with a tenfold molar excess of the spin radical MTSL [*S*-(1-oxyl-2,2,5,5-tetramethyl-2,5-dihydro-1*H*-pyrrol-3-yl)methyl methanesulfonothioate; Toronto Research Chemicals, Ontario, Canada] at 4°C overnight. Excess spin reagent was removed by gel-filtration chromatography in 20 m*M* HEPES, 150 m*M* NaCl pH 7.5. The spin-labelled samples were concentrated to 200 µ*M* for continuous-wave electron paramagnetic resonance (CW-EPR) experiments.

CW-EPR experiments were performed at the X-band (9.5 GHz) using a Bruker A300 spectrometer (Bruker Biospin GmbH, Rheinstetten, Germany) equipped with a high-sensitivity cavity (ER 4119HS; Bruker Biospin GmbH, Rheinstetten, Germany) at room temperature (298 K). Spectra were recorded at a microwave power of 2 mW over a scan width of 150 G with a field modulation of 1 G at a frequency of 100 kHz. Samples were placed in a glass capillary tube with a volume of approximately 25 µl. Data acquisition was performed 20 times to achieve a reasonable signal-to-noise ratio.

## 3. Results   

### 3.1. *Sa*_enolase exists as both an octamer and a dimer in solution   

**Figure 1:**
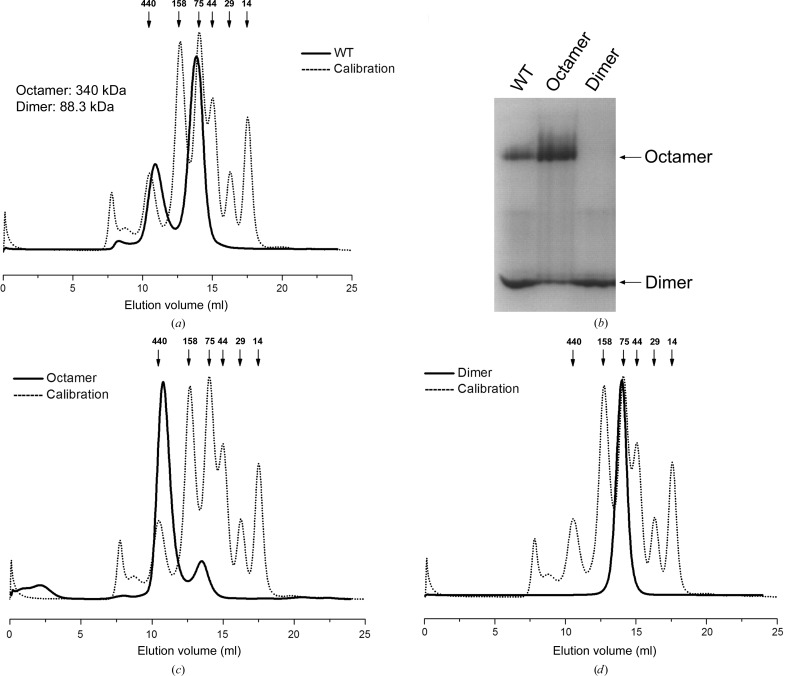
Size-exclusion chromatography of wild-type *Sa*_enolase. (*a*) Size-exclusion chromatography of *Sa*_enolase using a Superdex 200 16/60 column (GE). The molecular weights of the octamer and dimer peaks are calculated from the elution volume based on the standard curve. (*b*) Native PAGE analysis of *Sa*_enolase. The first lane (WT) is the sample before SEC; the second and third lanes correspond to the octamer and dimer peaks after SEC. Size-exclusion chromatographic analysis of the octameric (*c*) and dimeric (*d*) forms of *Sa*_enolase was performed using a Superdex 100 10/300 GL column (GE) to monitor the interconversion and equilibration of both forms. The chromatographic separation of the standard proteins is shown as a black dashed line and the theoretical molecular weights are shown above.

Enolase has been identified as a dimer in most organisms. However, octameric enolase has been observed in some bacterial species (Raghunathan *et al.*, 2014 ▸; Lu *et al.*, 2012 ▸; Ehinger *et al.*, 2004 ▸). *Sa*_enolase as presented here was observed as both a dimer and an octamer in solution (Fig. 1 ▸ *a*), a finding that has not previously been reported for other species. The two forms of *Sa*_enolase can be separated completely by size-exclusion chromatography (Fig. 1 ▸ *a*); the dimeric form is stable, while the octameric form partially disassembles to the dimeric form after storage at 4°C for 2 d (Figs. 1 ▸ *b*, 1 ▸ *c* and 1 ▸ *d*).

### 3.2. Overall structure   

To understand the difference between the octameric and dimeric forms of *Sa*_enolase, both forms were purified and crystallized for structure investigation. However, only an octameric structure was determined. The non-ligand-bound *Sa*_enolase crystals belonged to space group *P*42_1_2 and contained two monomers forming a single homodimer in the asymmetric unit. The *Sa*_enolase structure was solved by molecular replacement using the structure of *E. coli* enolase (PDB entry 1e9i) as a model. The structure of non-ligand-bound *Sa*_enolase was refined to 2.45 Å resolution with final *R*_work_ and *R*_free_ factors of 18.55 and 22.86%, respectively. Similar to previous structures, a single arginine residue, Arg400, resides in the disallowed region of the Ramachandran plot.

**Figure 2:**
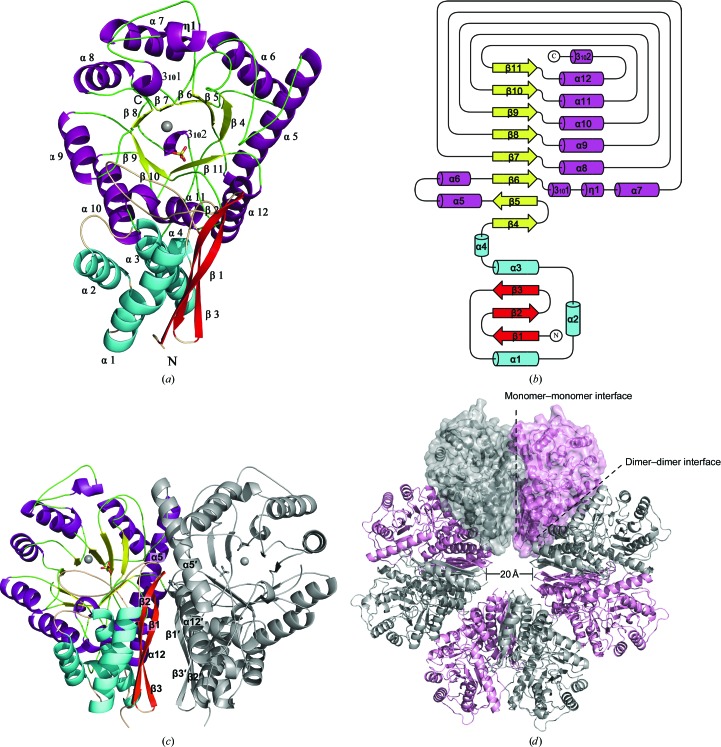
Overall structure of *Sa*_enolase. (*a*) Ribbon diagram of the overall structure of *Sa*_enolase. The secondary-structural elements are coloured cyan/red for the N-terminal domain and purple/yellow for the C-terminal barrel domain. The α-helices and β-strands are labelled in black. (*b*) Topology diagram of *Sa*_enolase. The secondary-structural elements are indicated. (*c*) The dimeric structure of *Sa*_enolase. Secondary-structure elements (β1–β3, α5 and α12) involved in dimerization are labelled in black. (*d*) The octameric structure of *Sa*_enolase. The monomers within a dimer are shown in grey and pink. The monomer–monomer interface and dimer–dimer interface are highlighted with black dashes.

The *Sa*_enolase monomer has two distinct domains, a small N-terminal domain (residues 1–137) and a large C-terminal barrel domain (residues 149–434), connected by a linker region (residues 138–148). The active site is located in the C-terminal domain and includes bound Mg^2+^ and sulfate ions (Fig. 2 ▸ *a*). The N-terminal domain comprises a three-stranded antiparallel β-sheet (β1–β3) followed by four α-helices (α1–α4). The C-terminal domain consists of eight β-strands (β4–β11) and eight α-helices (α5–α12), displaying an unusual eight-stranded α/β-barrel with β4–β11 arranged in an inner barrel-like structure surrounded by peripheral barrel walls comprised of α5–α12 (Fig. 2 ▸ *a*). In addition to the eight α-helices, there are three short helices included in the C-terminal domain: 3_10_1 and η1 are inserted between β6 and α7, while the third, 3_10_2, is located at the C-terminus. The topological features of the *Sa*_enolase barrel domain is ββαα(βα)_6_, which differs from the typical (βα)_8_ TIM-barrel topology (Banner *et al.*, 1975 ▸) in that β5 is antiparallel to the other β-strands and α5 is antiparallel to the rest of the helices (Fig. 2 ▸ *b*).

In the asymmetric unit, two monomers form a bufferfly-like dimer through interactions between β1–β3 and α5 and α12 from each monomer (Fig. 2 ▸ *c*). Structure alignment indicates that the two monomers are nearly identical, with a root-mean-square deviation (r.m.s.d.) of 0.2 Å for all C^α^ atoms. The four dimers pack together to form a ring-shaped octamer, the centre of which forms a small tunnel with a diameter of ∼20 Å (Fig. 2 ▸ *d*).

### 3.3. PEP- and Mg^2+^-binding site   

PEP-bound *Sa*_enolase crystals were obtained by the co-crystallization of *Sa*_enolase with the substrate 2-PG. The crystals belonged to space group *I*4, with two monomers forming a single homodimer in the asymmetric unit. The structure of PEP-bound *Sa*_enolase was solved by molecular replacement using the non-ligand-bound structure as a model and was refined to 1.60 Å resolution, with final *R*_work_ and *R*_free_ factors of 14.84 and 16.35%, respectively. A single arginine residue, Arg400, resides in the disallowed region of the Ramachandran plot.

**Figure 3:**
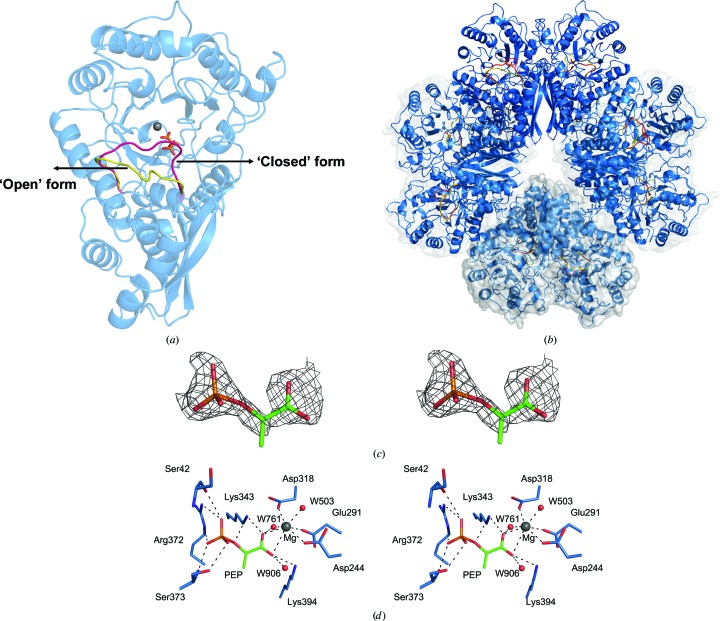
PEP-binding site of *Sa*_enolase. Ribbon diagrams of the dimeric structure (*a*) and the octameric structure (*b*) of the PEP-bound form of *Sa*_enolase. The two alternative conformations of catalytic loop 1 are highlighted in red for the closed form and yellow for the open form. PEP is shown as sticks. (*c*) Stereoview of the *F*_o_ − *F*_c_ difference electron-density map for PEP contoured at 2.5σ. The map was calculated with coefficients for a model in which the PEP was omitted. (*d*) Stereoview of the PEP- and Mg^2+^-binding sites. Active-site residues and PEP are depicted as sticks with C atoms coloured blue and green, respectively. Hydrogen bonds and metal-coordination bonds are shown as black dashed lines.

The structure of PEP-bound *Sa*_enolase is similar to the non-ligand-bound structure (with an overall r.m.s.d. of 0.3 Å for C^α^ atoms), with the exception of a conformational change involving catalytic loop 1 (L1; residues 38–64) in the PEP-bound structure (Figs. 3 ▸ *a* and 9). In contrast to L1, the other two identified catalytic loops (Wedekind *et al.*, 1994 ▸; Navarro *et al.*, 2007 ▸), loop 2 (L2; residues 154–163) and loop 3 (L3; residues 249–269), show no conformational change in the structures (Fig. 9). The PEP-bound structure also forms an octamer, with each monomer binding one PEP molecule and one Mg^2+^ ion (Fig. 3 ▸ *b*). The substrate 2-PG was used for co-crystallization; however, based on the *F*_o_ − *F*_c_ electron-density map the product PEP was included in the active site (Fig. 3 ▸ *c*). The PEP-binding site is located near the centre of the C-terminal barrel domain (Fig. 3 ▸ *a*). L1 exhibits two alternate arrangements, presenting both ‘open’ and ‘closed’ enzyme conformations (Fig. 3 ▸ *a*). The inter­actions of PEP with surrounding residues in the ‘closed’ conformation are illustrated in Fig. 3 ▸(*d*). Lys343, Lys394, Asp318, Arg372 and Ser373, together with Ser42 from L1, constitute this inter­action network. The PEP carboxyl group penetrates to the centre of the binding site by forming salt bridges to Lys343 and Lys394 (Fig. 3 ▸ *d*). On the opposite side, the PEP phosphate group forms hydrogen bonds to Arg372, Ser373 and Ser42. The O^1^ atom of the phosphate group forms hydrogen-bond contacts to the Ser373 side chain at a distance of 3.1 Å; O^2^ is stabilized by the Ser42 carbonyl and the N^η2^ atom of Arg372, and O^3^ forms hydrogen-bond contacts to the Ser373 amide N atom and the N^∊^ atom of Arg372 (Fig. 3 ▸ *d*). Additionally, the PEP O^2^ also forms hydrogen-bond contacts to the N^ζ^ atom of Lys343. The PEP-binding site in the ‘open’ conformation mimics that of the ‘closed’ conformation, but lacks interaction with Ser42 (data not shown). The residues participating in PEP interactions are conserved, indicating that similar enzymatic mechanisms exist across species (Supplementary Fig. S1).

Although enolase is a metalloenzyme containing two metal-binding sites in the active site, our structure only displays one bound Mg^2+^ ion near the PEP carboxyl group. As shown in Fig. 3 ▸(*d*), two PEP carboxyl O atoms occupy two positions within the Mg^2+^-binding site through coordinate bonds. The additional three sites are occupied by the carboxyl O atoms of Asp318, Asp244 and Glu291. The other two coordinating bonds are formed by two water molecules.

### 3.4. Assembly of the *Sa*_enolase octamer   

In contrast to other reported enolases, *Sa*_enolase exists as both an octamer and a dimer in solution (Fig. 1 ▸). However, only the octamer was observed in the crystal structure, regardless of the purified form used for screening. These findings prompted us to further explore the contact interfaces that mediate octamer assembly. As previously mentioned, the two *Sa*_enolase monomers in the asymmetric unit form a butterfly-like dimer. These subsequently form an octamer in which four dimers related by fourfold crystallo­graphic rotation symmetry pack against one another (Fig. 2 ▸ *d*).

**Figure 4:**
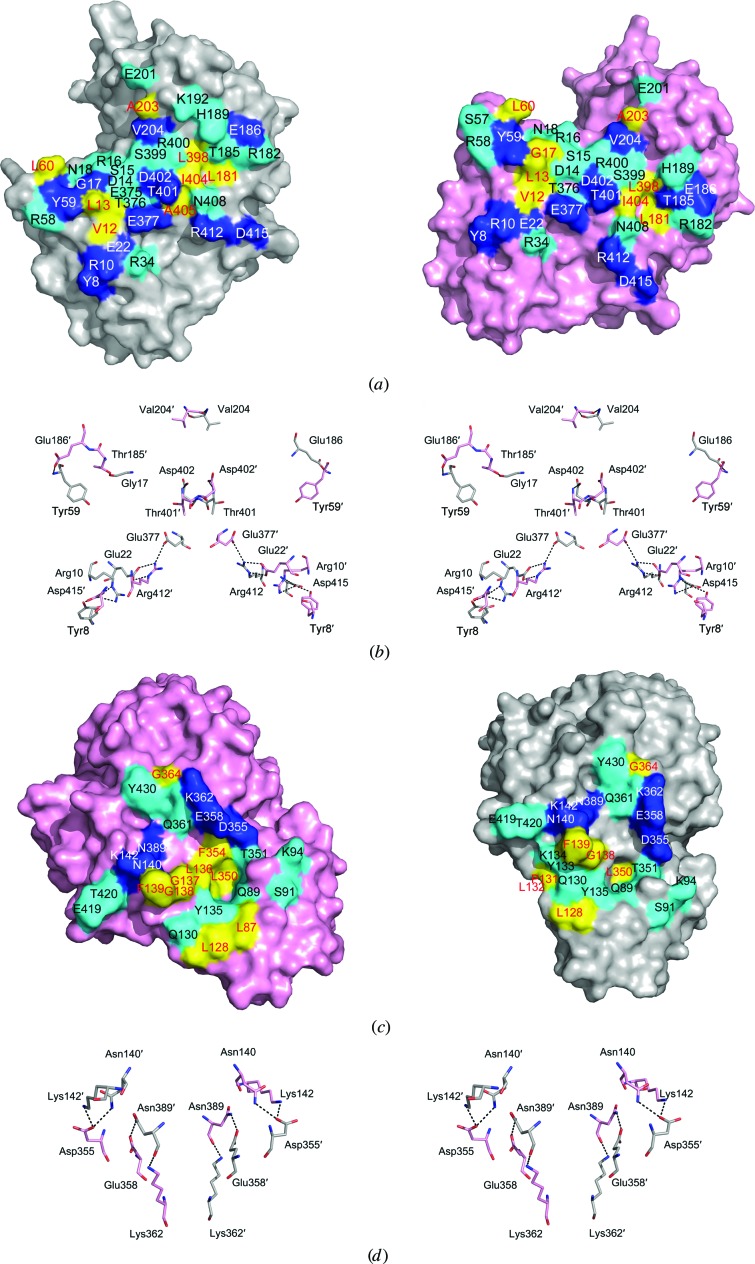
Interface analysis of *Sa*_enolase. (*a*) The monomer–monomer interface surfaces are shown as an open-book presentation. (*b*) A stereoview of the hydrogen-bond network at the monomer–monomer interface. Residues from the neighbouring monomer are indicated with primes. (*c*) An open-book presentation of the dimer–dimer interface. (*d*) A stereoview of the hydrogen-bond network at the dimer–dimer interface. Hydrogen-bond, hydrophilic and hydrophobic interactions are coloured blue, cyan and yellow, respectively. Residues involved in hydrogen-bond, hydrophilic and hydrophobic interactions are labelled in white, black and red, respectively.

The *Sa*_enolase octamer is formed by two types of interface: a monomer–monomer interface within the butterfly-like dimer in one asymmetric unit and a dimer–dimer interface between the butterfly-like dimers in the octamer ring (Fig. 2 ▸ *d*). The monomer–monomer interface is vast and has a buried surface area of 1747 Å^2^. An open-book view reveals that a total of 36 and 34 residues from each monomer are buried in the monomer–monomer interface (Fig. 4 ▸ *a*). Two monomers form a stable dimer *via* hydrogen-bond (blue), hydrophilic (cyan) and hydrophobic (yellow) interactions. 12 residues from each monomer form 19 hydrogen bonds across the dimer interface (Fig. 4 ▸ *b*). These residues are nearly identical between the respective monomers owing to the twofold noncrystallographic rotational symmetry exhibited by the dimer structure, with the exception of Gly17 from one monomer and Thr185 from the other. The twofold noncrystallographic rotation symmetry axis-related residues form nine pairs of hydrogen bonds, while the other two residues Gly17 and Thr185′ form the last one (Fig. 4 ▸ *b*).

The dimer–dimer interfaces within the octamer form along the outer edges of neighbouring butterfly-like dimers and result in a buried surface area of ∼1287 Å^2^. As shown in Figs. 4 ▸(*c*) and 4 ▸(*d*), 25 residues from each monomer are involved in the dimer–dimer interfaces, forming eight hydrogen bonds (blue) and multiple hydrophilic (cyan) and hydrophobic (yellow) interactions. Since the two neighbouring molecules in the dimer–dimer interface are related by a fourfold axis of symmetry, the residues buried at the interface are also nearly identical to those in the two respective monomers, especially the six residues involved in hydrogen bonds (Figs. 4 ▸ *c* and 4 ▸ *d*). A hydrophobic core formed by residues Leu136–Phe139, Leu350 and Phe354 on the surface of one dimer accommodates the phenyl group of Phe139 from the neighbouring dimer. Nine polar residues, Ser91, Lys94, Thr351, Gln89, Tyr135, Gln130, Tyr133, Thr420 and Thr419, are arranged along the surface of both monomers and interact with each other bordering the dimer–dimer interface. Additionally, eight hydrogen bonds formed by Asn140, Lys142, Asp355, Glu358, Lys362 and Asn389 from both monomers strengthen the dimer–dimer interaction (Figs. 4 ▸ *c* and 4 ▸ *d*). Residues involved in forming the hydrophobic core and hydrogen bonds are conserved among octameric enolases, confirming their importance in the dimer–dimer interactions (Figs. 4 ▸ *c* and 4 ▸ *d* and Supplementary Fig. S1).

### 3.5. Oligomerization of wild-type *Sa*_enolase and mutants   

**Figure 5:**
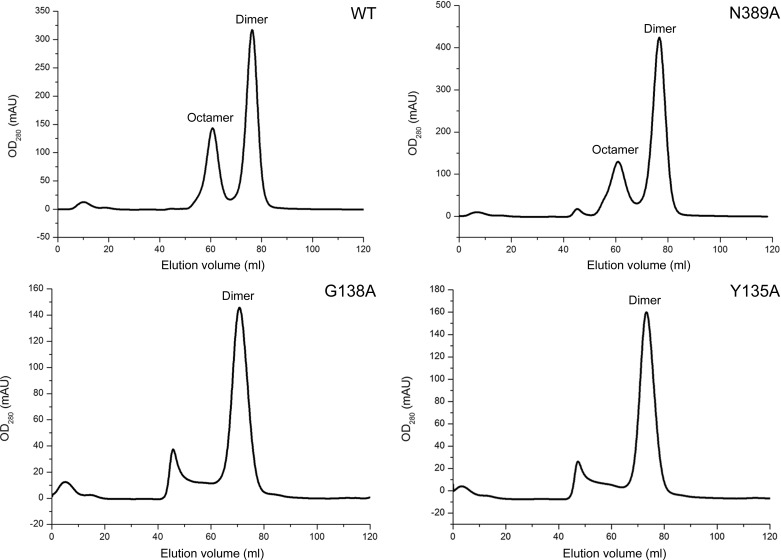
Oligomeric state analysis of wild-type and mutants of *Sa*_enolase using size-exclusion chromatography with a Superdex 200 16/60 column (GE).

Enolase typically exists as a homodimer in eukaryotes and most prokaryotes, while some bacterial enolases have been reported to be octamers. Here, we observe both octameric and dimeric forms of *Sa*_enolase in solution (Fig. 1 ▸). The interfaces associated with octameric *Sa*_enolase prompted us to analyze the amino acids involved in dimer–dimer interactions. As discussed, ∼36 residues from one monomer are involved in the monomer–monomer interface, while 25 residues contribute to the dimer–dimer interaction. Interestingly, there is no overlap between monomer–monomer interface residues and dimer–dimer interface residues (Fig. 4 ▸). Multiple sequence alignment of *Sa*_enolase against other octameric enolases reveals that most of the residues involved in the dimer–dimer interface are highly or partially conserved in octameric enolases, including residues Tyr135–Asn140 at the centre of the interface and Asp355, Glu358 and Asn389 involved in hydrogen-bond interactions (Supplementary Fig. S1). In order to analyze the oligomeric state of *Sa*_enolase, the molecular weights of the wild-type protein and six mutants (Y135A, G138A, F139A, N140A, D355A and N389A) were estimated by size-exclusion chromatography. As shown in Fig. 5 ▸ and Supplementary Fig. S2, wild-type *Sa*_enolase exists as both dimers and octamers in solution and the N389A mutation only slightly disassembles the octamer. In addition, N140A, F139A or D355A mutations significantly impair the formation of the octamer, while no octameric form of the Y135A and G138A mutants was observed in solution. Our studies indicate that the conserved interface residues are critical for octamer formation.

### 3.6. Catalytic activity of the dimer and octamer   

**Figure 6:**
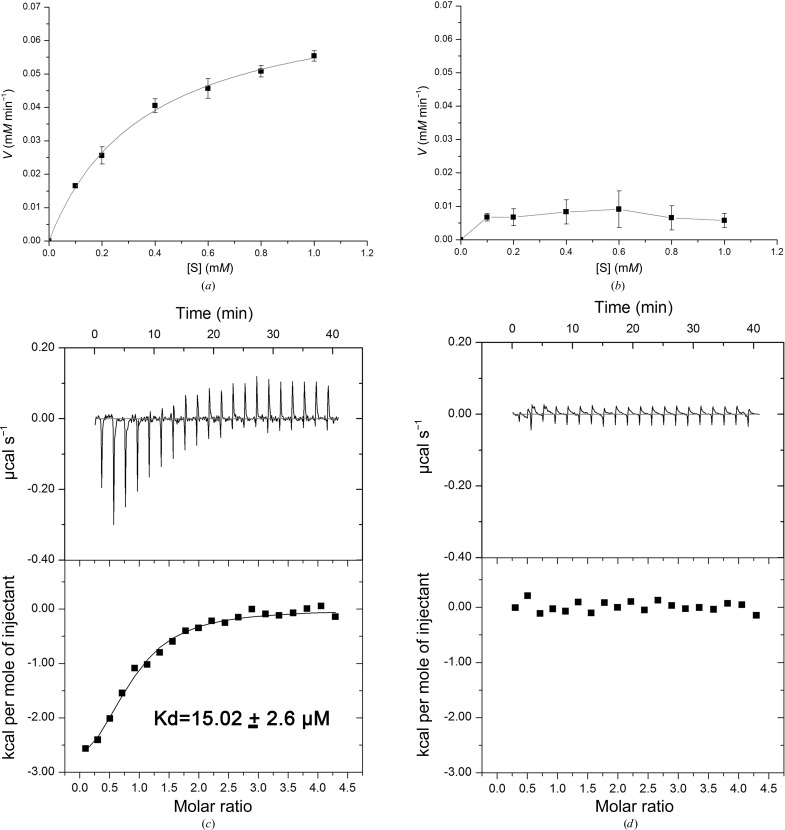
Activity and ITC assays of octameric (*a*, *c*) and dimeric (*b*, *d*) *Sa*_enolase.

Given that *Sa*_enolase exists as an octamer in both the crystal structure and in solution, the octameric form may constitute the biological unit for catalytic activity. To test this hypothesis, we determined the enzymatic activities of both the dimeric and the octameric enzymes. As expected, the *Sa*_enolase octamer exhibits a catalytic activity similar to the enolase activity reported for other species (Fig. 6 ▸ *a*), with a *K*_m_ of 0.37 m*M* and a *k*_cat_/*K*_m_ of 2.27 × 10^5^ *M* ^−1^ s^−1^ (Table 2 ▸). In contrast, the dimeric form appears to be catalytically inactive (Fig. 6 ▸ *b*). For further investigation, we used ITC assays to measure the binding of dimeric and octameric *Sa*_enolase to 2-PG. The ITC results indicated that the *Sa*_enolase octamer shows binding to 2-PG with a *K*_d_ value of 15.02 ± 2.6 µ*M* (Fig. 6 ▸ *c*), while the interaction between *Sa*_enolase and 2-PG is too weak to be detected by ITC assays (Fig. 6 ▸ *d*). These findings suggest that the octamerization of *Sa*_enolase is required for substrate binding and further prove that the octameric form is the functional unit for the catalytic activity of *Sa*_enolase.

### 3.7. Dynamics of catalytic loop L1   

**Figure 7:**
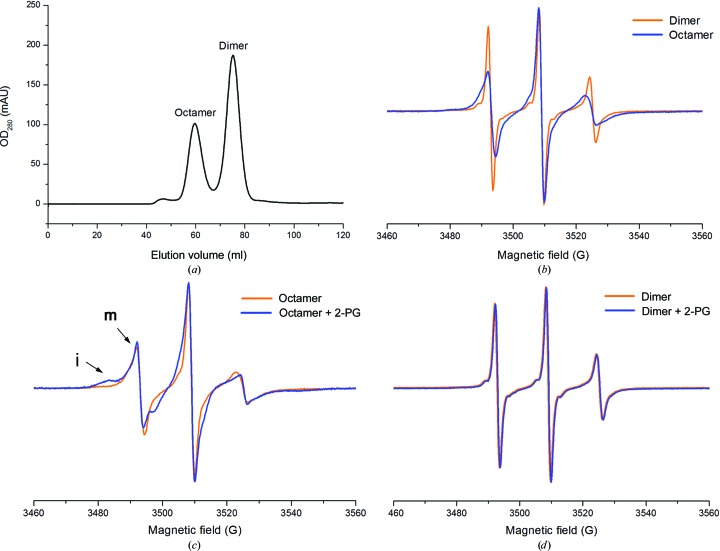
EPR spectra of spin-labelled T43C on catalytic loop 1 of the *Sa*_enolase octamer and dimer. (*a*) Size-exclusion chromatography of the *Sa*_enolase T43C/C245S mutant using a Superdex 200 16/60 column (GE). (*b*) EPR spectra of the T43C/C245S dimer and octamer without 2-PG. EPR spectra of the T43C/C245S octamer (*c*) and dimer (*d*) in the presence or absence of 2-PG. Each spectrum was normalized by the height of the central peak. ‘i’ and ‘m’ represent the ‘immobile’ and ‘mobile’ components, respectively.

Since the catalytic loop L1 is involved in substrate binding, we analyzed the dynamics of L1 in the *Sa*_enolase dimer and octamer by CW-EPR spectroscopy. CW-EPR spectroscopic experiments were performed for the *Sa*_enolase dimer and octamer in the presence and absence of 2-PG. To construct the single cysteine mutation for CW-EPR, the cysteine at site 245 was first mutated to serine and the Thr43 in L1 (residue 38–64) was then mutated to cysteine. The purified dimer and octamer of *Sa*_enolase T43C/C245S were separated by SEC (Fig. 7 ▸ *a*) and labelled with the spin radical MTSL through disulfide-bond formation between MTSL and the T43C residue. As shown in Fig. 7 ▸(*b*), apparent spectral broadening was observed when the *Sa*_enolase dimer packed into an octamer in the absence of 2-PG, indicating that the dynamic motion of the spin label L1 in the dimer is faster than in the octamer. After the addition of the substrate 2-PG, the CW-EPR spectra exhibited multiple motion components in the octamer, which strongly indicate the presence of both immobilized (i) and mobilized (m) components (Fig. 7 ▸ *c*). The two motional components detected in the CW-EPR spectra suggest two different motional or conformational states of L1 in the *Sa*_enolase octamer, which is consistent with our structural results that the immobilized component could be the ‘closed’ form stabilized by ligand binding. In contrast, the CW-EPR spectra of the *Sa*_enolase dimer remained unchanged after the addition of 2-PG (Fig. 7 ▸ *d*), which indicates that L1 in the dimer makes no response to the substrate. The different dynamic motion of L1 in the dimer may be the reason why the dimeric form of *Sa*_enolase is unable to bind the substrate. In addition to ITC assays, the CW-EPR data provide further evidence for our hypothesis that the octamerization of *Sa*_enolase is required for substrate binding and that the function of the protein is likely to be related to its quaternary structure.

## 4. Discussion   

### 4.1. Structural comparison with other enolases   

**Figure 8:**
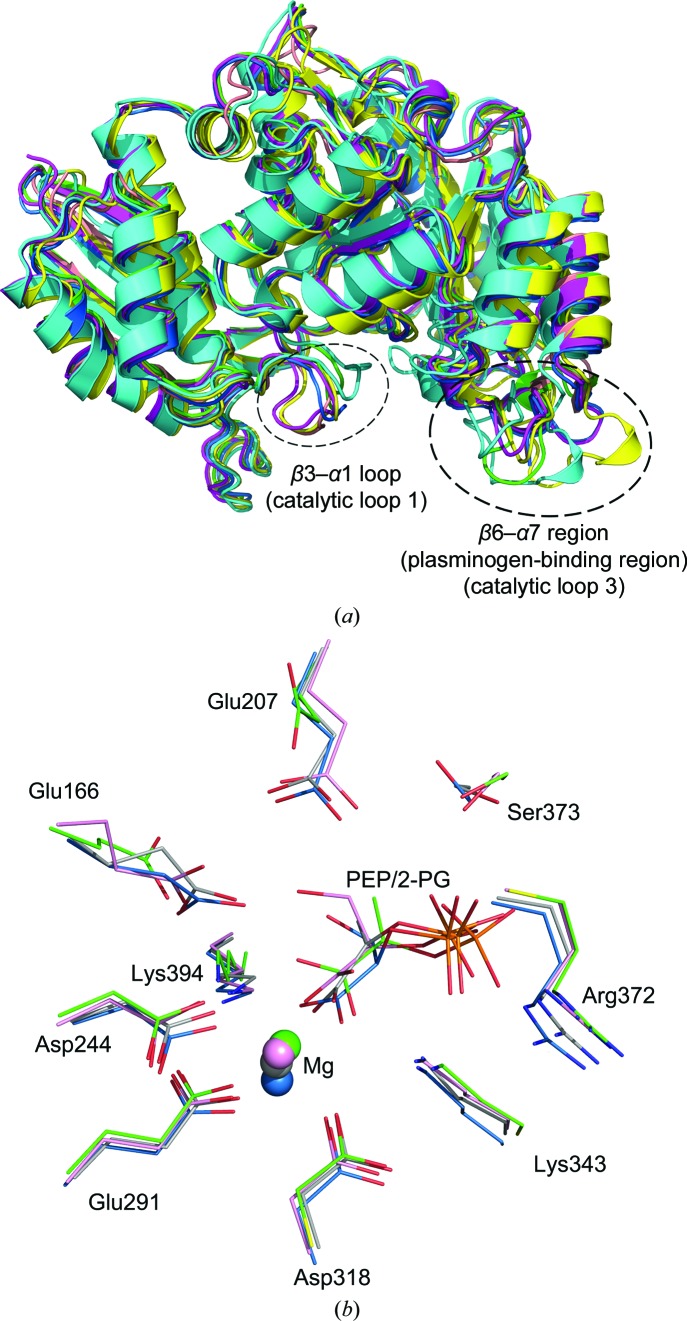
Structural comparison of *Sa*_enolase with other enolases. (*a*) Overall structure comparison of *Sa*_enolase with homologous structures from other species. Two regions that display conformational variations are marked with black dashed circles. Structures are coloured purple for *Sa*_enolase, pink for PDB entry 1e9i (*E. coli*), sky blue for PDB entry 4ewj (*S. pneumoniae*), green for PDB entry 1ebh (*S. cerevisiae*) and cyan for PDB entry 3b97 (*H. sapiens*). (*b*) Structural comparison of the ligand-binding site of *Sa*_enolase (green) with PDB entry 3ujf (sky blue), PDB entry 3qtp (pink) and PDB entry 2xgz (grey). Active-site residues and 2-­PG/PEP are depicted as sticks and labelled in black (*Sa*_enolase numbering). The Mg^2+^ ions are shown as spheres.

A structure-similarity search for *Sa*_enolase was performed using the *DALI* online server (Holm & Rosenström, 2010 ▸). The structures displaying the greatest similarity are enolase orthologues from either prokaryotic or eukaryotic species. In order to compare the *Sa_*enolase structure with other orthologues, enolases from representative species were selected for superimposition. As displayed in Fig. 7 ▸, *Sa*_enolase aligns well with orthologues from *Homo sapiens* (PDB entry 3b97; Kang *et al.*, 2008 ▸), *Saccharomyces cerevisiae* (PDB entry 1ebh; Wedekind *et al.*, 1995 ▸), *Enterococcus hirae* (PDB entry 1iyx; Hosaka *et al.*, 2003 ▸), *Streptococcus pneumoniae* (PDB entry 1w6t; Ehinger *et al.*, 2004 ▸) and *E. coli* (PDB entry 1e9i; Kuhnel & Luisi, 2001 ▸). Among these, *S. pneumoniae* enolase shares the highest structural similarity to *Sa*_enolase, with a *Z*-score of 67.8 and an all-C^α^-atom r.m.s.d. of 0.4 Å, while the remainder deviate on C^α^ superposition with r.m.s.d. values ranging from 0.6 to 1.8 Å. Although the overall enolase structure is highly conserved, conformational variations can be observed in two regions. One is in the loop located between β3 and α1, which is referred to as L1 and is involved in substrate binding (Wedekind *et al.*, 1994 ▸). The other is the random coil bridging β6 and α7, which is referred to as L3 and forms the plasminogen-binding motif (Ehinger *et al.*, 2004 ▸). Both of these two functionally related loops are flexible in our structure (Fig. 8 ▸ *a*).

Aside from non-ligand-bound forms of enolase, some 2-PG/PEP-bound structures also exist. The structures of enolases in the presence or absence of 2-PG/PEP maintain similarity, except where L1 displays either an ‘open’ or ‘closed’ conformation (Navarro *et al.*, 2007 ▸; Larsen *et al.*, 1996 ▸). Here, we compare the active sites of structures displaying the ‘closed’ conformation. As shown in Fig. 8 ▸(*b*), comparison of the PEP-binding site of *Sa*_enolase with those of *H. sapiens* (PDB entry 3ujf; Qin *et al.*, 2012 ▸), *Entamoeba histolytica* (PDB entry 3qtp; Schulz *et al.*, 2011 ▸) and *S. cerevisiae* (PDB entry 2xgz; Schreier & Hoecker, 2010 ▸) reveals a highly conserved active-site orientation around PEP and Mg^2+^. The superimposition of the ligands 2-PG/PEP and Mg^2+^ displays little variation and the residues involved in PEP/2-PG and Mg^2+^ binding are highly conserved in all four structures. Two glutamate residues, Glu166 and Glu207, that interact with the 2-PG hydroxyl group in the 3ujf and 3qtp structures are also conserved in *Sa*_enolase and the 2xgz structure (Supplementary Fig. S1). These results indicate that *Sa*_enolase is structurally similar to other enolases.

### 4.2. Structure comparison of the PEP-bound variant with the non-ligand-bound variant   

**Figure 9:**
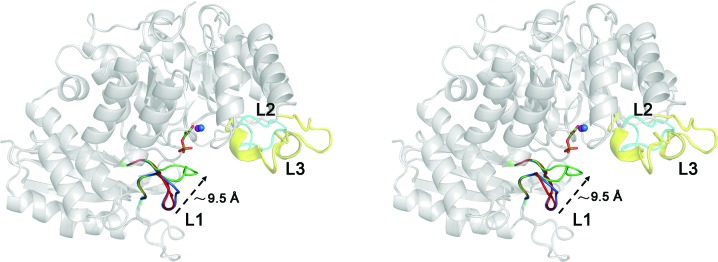
Superposition of the non-ligand-bound form and the PEP-bound form of *Sa*_enolase shown as a stereoview. The catalytic loop L1 in the non-ligand-bound form is coloured blue. The ‘open’ and ‘closed’ forms of the catalytic loop L1 in the PEP-bound structure are coloured red and green, respectively. The catalytic loops L2 and L3 are coloured cyan and yellow, respectively. PEP is shown as sticks. Mg^2+^ ions are coloured blue for the non-ligand-bound structure and purple for the PEP-bound structure. The rest of the structure is coloured grey. The distance corresponding to the conformational change of catalytic loop 1 is labelled in black.

Compared with the non-ligand-bound form, there are few enolase structures available with substrate/product bound. Yeast enolase folds into different conformations in the presence or absence of PEP, with the structures described as ‘open’ (PDB entry 1ebh; Wedekind *et al.*, 1995 ▸) and ‘closed’ (PDB entry 2one; Zhang *et al.*, 1997 ▸). Structural comparison of non-ligand-bound *Sa*_enolase with yeast enolase reveals that the non-ligand-bound *Sa*_enolase presented here assumes a conformation similar to the ‘open’ conformation of yeast enolase. Interestingly, L1 in the PEP-bound structure of *Sa*_enolase displays both the ‘open’ and ‘closed’ conformations. Superimposition of the non-ligand-bound and PEP-bound structures of *Sa*_enolase reveals that conformational changes occur following ligand binding. The primary difference involves the orientation of L1, with residues 36–44 moving approximately 9.5 Å towards the centre of the active site in order to cover the catalytic pocket (Fig. 9 ▸). Occupation of the binding site by a sulfate ion (in the non-ligand-bound structure) instead of the PEP phosphate group (in the PEP-bound structure) cannot trigger the conformational change of L1, indicating that the conformational change only occurs following substrate binding (Fig. 9 ▸). These structural studies demonstrate that the flexible catalytic L1 undergoes significant conformational changes during substrate binding, enzymatic catalysis and product release. These translate to allowing the cavity to ‘open’ in order to allow substrate entrance, ‘close’ in order to trigger the reaction and likely ‘open’ again in order to release the product. In contrast to the dynamic motion of L1, the motions of the other catalytic loops L2 and L3 are comparatively subtle. To the best of our knowledge, the structure of the PEP-bound form of *Sa*_enolase constitutes the first Gram-positive bacterial structure solved with ligand bound and displaying evidence of catalytic loop shifting within the active site.

### 4.3. The octameric form is the functional unit of *Sa*_enolase required for catalysis   

It has been reported that enolase exists either as a dimer or an octamer in solution; thus, both dimeric and octameric forms have been identified as the functional unit of enolase. Here, we report the existence of both dimeric and octameric forms of *Sa*_enolase in solution (Fig. 1 ▸). Enzymatic activity assays revealed that the octamer is able to catalyze the dehydration of 2-PG to yield PEP, indicating that octameric *Sa*_enolase is functional *in vitro* and likely also *in vivo* (Fig. 6 ▸ *a*, Table 2 ▸). However, the dimeric enzyme appeared to be catalytically inactive (Fig. 6 ▸ *b*, Table 2 ▸). The N389A mutant retains the ability to form both dimers and octamers in solution, yet only the octameric form is active (Fig. 5 ▸ and Supplementary Fig. S3). Additionally, only the dimeric form is observed for the Y135A and G138A mutants (Fig. 5 ▸), and these two mutants are also catalytically inactive (Supplementary Fig. S3). These results indicate that the dimeric form of *Sa*_enolase is unable to catalyze the dehydration of 2-PG. Moreover, the ITC results showed that the interaction of 2-PG with *Sa*_enolase dimers, including the wild-type dimer (Fig. 6 ▸ *d*), the F139A mutant and the D355A mutant (Supplementary Fig. S4), was too weak to be detected, which further indicated that the octamerization of *Sa*_enolase could be essential for substrate binding and strongly supported the octameric *Sa*_enolase as the functional unit for the 2-PG dehydration activity.

As discussed, *Sa*_enolase is structurally similar to other enolases, indicating a shared catalytic mechanism. The enolase active site is located within a single subunit and the monomeric enolase is catalytically active (Holleman, 1973 ▸). Therefore, the question as to why both dimeric and octameric forms of *Sa*_enolase exist in solution, yet only the octamer is able to bind substrate and be catalytically active, remains open. To explore the relationship between the quaternary structure and the enzyme function of *Sa*_enolase, mobility analysis of L1 using CW-EPR spectroscopy was performed on the *Sa*_enolase dimer and octamer in the presence or absence of 2-PG. The CW-EPR results indicate that the dynamic motion of L1 in the dimer is faster than in the octamer and that L1 in the dimer is unable to be stabilized by the addition of substrate (Fig. 7 ▸). These findings indicate that the enzymatic activity of *Sa*_enolase seems to correlate with its quaternary structure.

Eukaryotic enolases have been reported to exist as dimers, while prokaryotic enolases exist either as dimers or octamers, with the exception of *Sa*_enolase, which exists in both forms. Since only a small number of octameric enolases have been identified and some of the observed octamers may not have beeen fully investigated with regard to oligomerization state, it is difficult to determine whether *Sa*_enolase constitutes the only evidence of the enzyme existing as both a dimer and an octamer. Multiple sequence alignment and dimer–dimer interface analyses of *Sa*_enolase and other enolases indicated that several residues buried at the dimer–dimer interface are only conserved in species in which octameric forms of enolase exist (Supplementary Fig. S1 and Fig. 4 ▸ *c*). Eukaryotic enolases lack these conserved dimer–dimer interface residues, which may result in the formation of only dimeric enolase (Supplementary Fig. S1).

Enolase is well known as a phosphopyruvate hydratase that catalyses the conversion of 2-PG to PEP in the glycolysis pathway. Recently, enolase has been identified to have moonlighting functions in which it interacts with plasminogen (Mölkänen *et al.*, 2002 ▸) and binds laminin on the surface of *S. aureus* (Carneiro *et al.*, 2004 ▸). In addition, enolase has also been characterized as a component of the mRNA degradosome in *B. subtilis* (Lehnik-Habrink *et al.*, 2010 ▸) and *S. aureus* (Roux *et al.*, 2011 ▸). Given that enolase is a multi-functional enzyme with different localizations and that it interacts with different proteins, the octameric and dimeric forms of *Sa*_enolase may both be functional in different biological processes. Our recent studies showed that the *Sa*_enolase dimer may be involved in interaction with some mRNA degradosome components; however, further *in vitro* and *in vivo* evidence is required for further investigation.

## Supplementary Material

PDB reference: *Sa*_enolase, 5bof

PDB reference: complex with PEP, 5boe

Supporting Information.. DOI: 10.1107/S1399004715018830/qh5032sup1.pdf

## Figures and Tables

**Table 1:** Data-collection and refinement statistics Values in parentheses are for the highest shell.

	*Sa*_enolase	*Sa*_enolase–PEP
Data collection		
Space group	*P*42_1_2	*I*4
Unit-cell parameters		
*a* = *b* (Å)	164.7	145.15
*c* (Å)	77.3	100.51
α = β = γ (°)	90	90
Resolution range (Å)	50–2.45 (2.54–2.45)	50–1.60 (1.63–1.60)
No. of unique reflections	39128	136548
Wilson plot *B* factor (Å^2^)	38.5	11.2
*R*_meas_ † (%)	8.6 (51.9)	9.2 (49.8)
Mean *I*/σ(*I*)	29.2 (4.5)	40.3 (6.8)
Completeness (%)	98.3 (99.4)	100.0 (100.0)
Multiplicity	7.3 (7.4)	8.2 (8.1)
Refinement		
Resolution range (Å)	50–2.45	50–1.60
*R*_work_ ‡/*R*_free_ § (%)	18.55/22.86	14.84/16.35
R.m.s. deviations		
Bond lengths (Å)	0.008	0.009
Bond angles (°)	1.286	1.376
*B* factors (Å^2^)		
Protein	45.97	15.46
Water	36.01	26.73
Mg^2+^	33.74	11.12
Other ligands	59.36	29.52
Ramachandran plot		
Most favoured regions (%)	97.5	97.8
Additionally allowed regions (%)	2.3	2.0
Outliers (%)	0.2	0.2

† *R*_meas_ was estimated by multiplying the conventional *R*_merge_ value by the factor [*N*/(*N* − 1)]^1/2^, where *N* is the data multiplicity; *R*_merge_ = 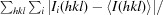 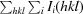, where *I_i_*(*hkl*) is the intensity of the *i*th measurement and 〈*I*(*hkl*)〉 is the mean intensity for that reflection.

‡ *R* = 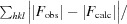 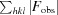, where |*F*_obs_| and |*F*_calc_| are the observed and calculated structure-factor amplitudes, respectively.

§ *R*_free_ was calculated with 5.0% of the reflections in the test set.

**Table 2:** Kinetic constants of the *Sa*_enolase octamer and dimer

	*V*_max_ (*M* min^−1^)	*K*_m_ (*M*)	*k*_cat_ (s^−1^)	*k*_cat_/*K*_m_ (*M* ^−1^ s^−1^)
Octamer	0.748 ± 0.0241 × 10^−4^	0.365 ± 0.030 × 10^−3^	0.83 × 10^2^	2.27 × 10^5^
Dimer	ND†	ND	ND	ND

† Not detectable.
