# Supplementary material for: Octameric structure of Staphylococcus aureus enolase in complex with phosphoenolpyruvate
Source: Acta Crystallogr D Biol Crystallogr. 2015 Nov 26;71(Pt 12):2457–70. doi: 10.1107/S1399004715018830 (PMC4667285; doi:10.1107/S1399004715018830)
Supplement: Supplementary file 1 [file d-71-02457-sup1.pdf]

Acta Cryst D

BIOLOGICAL
CRYSTALLOGRAPHY

Volume 71 (2015)

Supporting information for article:

Octameric structure of *Staphylococcus aureus* enolase in complex with phosphoenolpyruvate

Yunfei Wu, Chengliang Wang, Shenglong Lin, Minhao Wu, Lu Han, Changlin Tian, Xuan Zhang and Jianye Zang

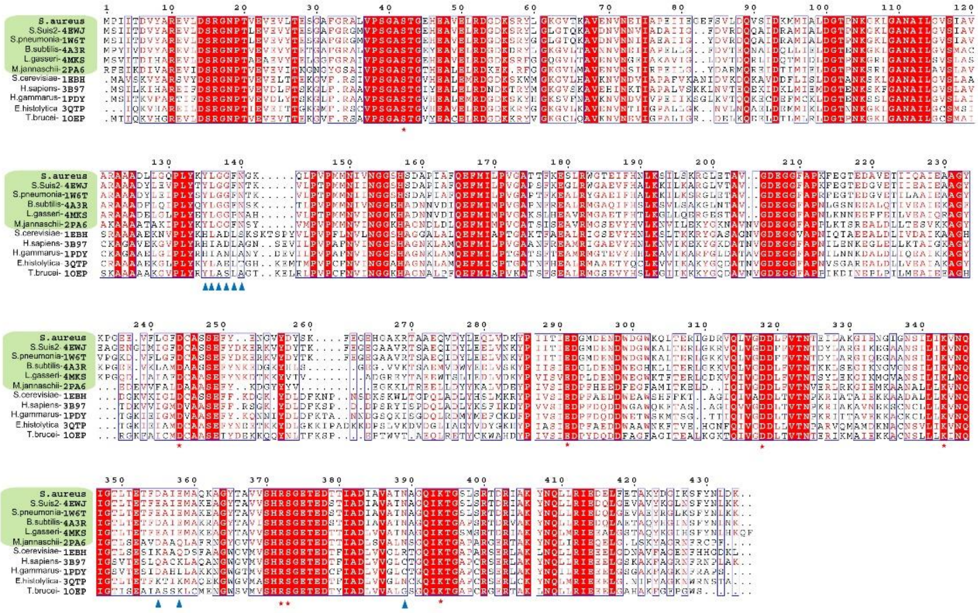

Figure S1 Multiple sequences alignment of enolase from representative species. Enolases from S. suis 2, S. pneumoniae, B. subtilis, L. gasseri, M. jannaschii, S. cerevisiae, H. sapiens, H. gammarus, E. histolytica, and T. brucei are used for sequences alignment. Active site residues are marked with red star, conserved dimer-dimer interface residues are marked with blue triangle. The octamric enolases are highlighted with green background

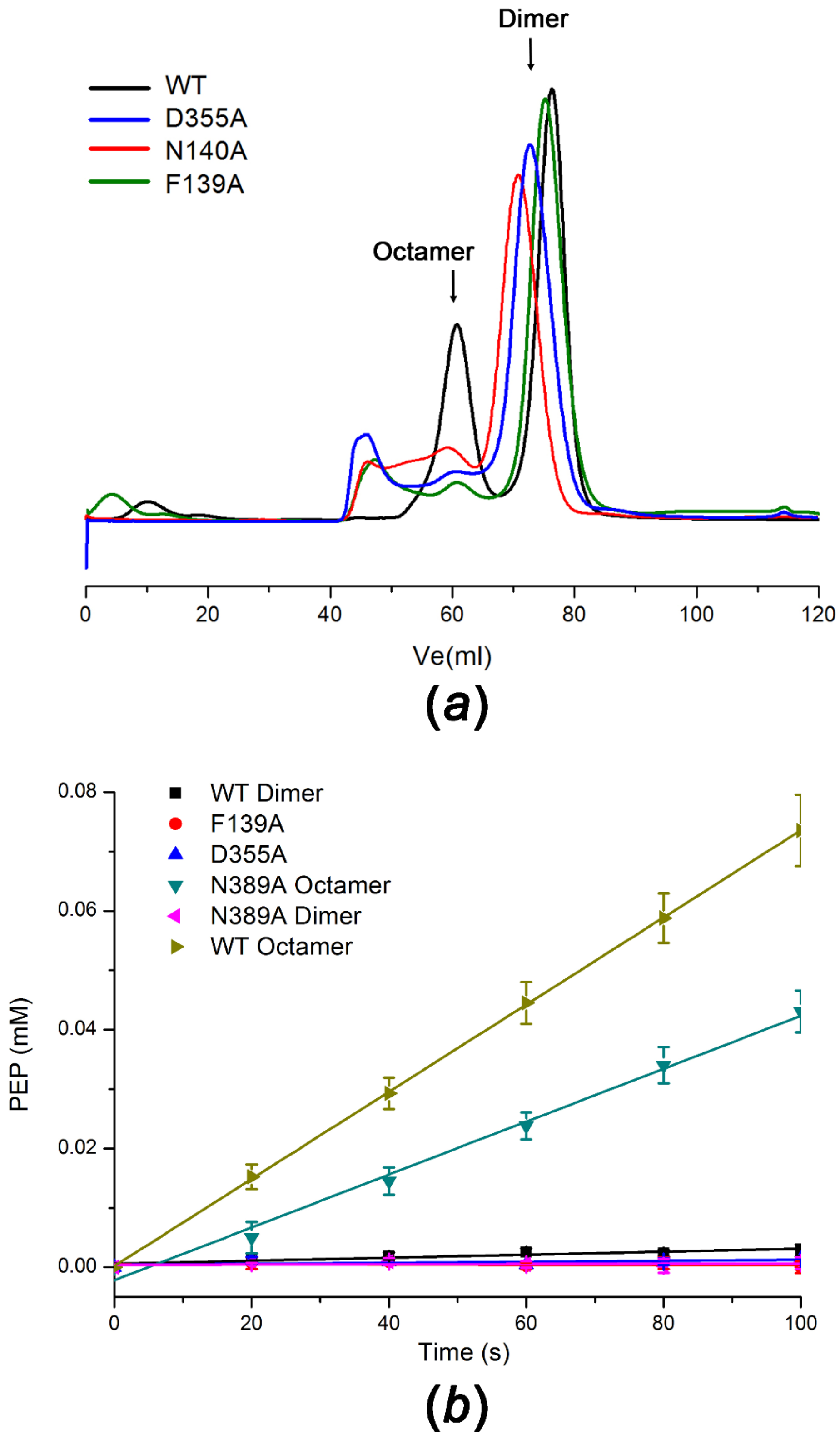

Figure S2 Oligomeric states analysis and enzymatic activity assays of WT *Sa*_enolase and its mutants. (*a*) Oligomeric states analysis of wild type *Sa*_enolase and its mutants using size exclusion chromatography with column Superdex200 16/60 (GE). (*b*) Activity assays were

performed in 20 mM IMD/HCl, 400 mM KCl, 1 mM $Mg(Ac)_2$, pH 7.0 buffer using 30 nM enzyme and 1 mM 2-PG to a final volume of 100 µL. N389A octamer had partial enzymatic activity comparing to WT octamer, while WT dimer, F139A, D355A, and N389A dimer are inactive. Each reaction was repeated for three times.

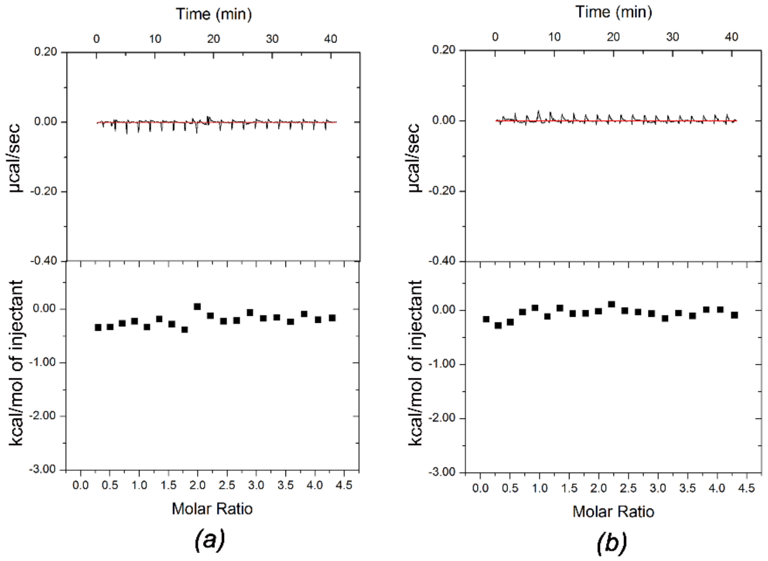

Figure S3 ITC assays for *Sa*_enolase mutants F139A (*a*) and D355A (*b*) binding to 2-PG.
